# The Potential of Multi-Biomarker Panels in Nutrition Research: Total Fruit Intake as an Example

**DOI:** 10.3389/fnut.2020.577720

**Published:** 2021-01-14

**Authors:** Aoife E. McNamara, Janette Walton, Albert Flynn, Anne P. Nugent, Breige A. McNulty, Lorraine Brennan

**Affiliations:** ^1^School of Agriculture and Food Science, UCD Institute of Food and Health, University College Dublin, Dublin, Ireland; ^2^UCD Conway Institute of Biomolecular and Biomedical Research, University College Dublin, Dublin, Ireland; ^3^School of Food and Nutritional Sciences, University College Cork, Cork, Ireland; ^4^Department of Biological Sciences, Cork Institute of Technology, Cork, Ireland; ^5^School of Biological Sciences, Institute for Global Food Security, Queens University Belfast, Belfast, United Kingdom

**Keywords:** dietary biomarkers, nutrition, dietary assessment, food intake, metabolomics, multi-biomarker panel

## Abstract

Dietary and food intake biomarkers offer the potential of improving the accuracy of dietary assessment. An extensive range of putative intake biomarkers of commonly consumed foods have been identified to date. As the field of food intake biomarkers progresses toward solving the complexities of dietary habits, combining biomarkers associated with single foods or food groups may be required. The objective of this work was to examine the ability of a multi-biomarker panel to classify individuals into categories of fruit intake. Biomarker data was measured using ^1^H NMR spectroscopy in two studies: (1) An intervention study where varying amounts of fruit was consumed and (2) the National Adult Nutrition Survey (NANS). Using data from an intervention study a biomarker panel (Proline betaine, Hippurate, and Xylose) was constructed from three urinary biomarker concentrations. Biomarker cut-off values for three categories of fruit intake were developed. The biomarker sum cut-offs were ≤ 4.766, 4.766–5.976, >5.976 μM/mOsm/kg for <100, 101–160, and >160 g fruit intake. The ability of the biomarker sum to classify individuals into categories of fruit intake was examined in the cross-sectional study (NANS) (*N* = 565). Examination of results in the cross-sectional study revealed excellent agreement with self-reported intake: a similar number of participants were ranked into each category of fruit intake. The work illustrates the potential of multi-biomarker panels and paves the way forward for further development in the field. The use of such panels may be key to distinguishing foods and adding specificity to the predictions of food intake.

## Introduction

Dietary assessment is important for the elucidation of diet-disease relationships; however, traditional dietary assessment techniques are subject to some well-documented limitations (1). The identification of dietary and food intake biomarkers offers the potential of accurate and objective measure of food intake (2, 3). Food intake biomarkers are single metabolites, or a combination of metabolites, reflecting the consumption of either a specific food or food group, displaying a clear time- and dose-response after intake (4). An extensive range of putative intake biomarkers of commonly consumed foods have been identified to date (5) but more work is needed toward confirming the utility of such biomarkers. To be efficient indicators of dietary intake, biomarkers need to be validated including analysis of sensitivity, specificity, and dose-response (6). Selecting a single intake biomarker to represent exact dietary intake is difficult due to the overlapping range of nutrients, non-nutrients, and bioactives present in foods. However, previously investigated food intake biomarkers have proven to be good predictors of consumption for classes within food groups, such as fruits and vegetables (7). As the field of food intake biomarkers progresses, and the need to elucidate the complexities of dietary habits arises, combining biomarkers associated with single foods or food groups may be required (8). It is possible that by combining two or more biomarkers together into multi-biomarker panels could result in more sensitive and specific estimates of intake.

Development of validated and comprehensive multi-biomarker panels have the potential to add value to the assessment of dietary intake by enabling the capture of a broad range of dietary exposure including bioactive compounds, foods, food groups, and complex dietary patterns (9). Multi-biomarker panels have been previously used to classify individuals into banana consumers and non-consumers more robustly compared to individual biomarkers (10). In another example a multi biomarker panel, composed of beer ingredient and food processing biomarkers was capable of distinguishing beer consumption from urine samples collected before and up to 12 h after intake of beer with excellent specificity and sensitivity (11). Other examples of successful use of multiple biomarkers include the SU.VI.MAX study, where the sum of urinary flavonoid biomarkers demonstrated higher correlations with fruit and fruit juice consumption than any of the included biomarkers individually (12). A biomarker panel containing the wine biomarkers ethyl glucuronide and tartrate outperformed individual markers when predicting wine consumers and non-consumers [90.7% receiver operating characteristics (ROC) curves area under the curve (AUC) compared to 86.3% for ethyl glucuronide and 85.7% for tartrate]. This panel was validated in an epidemiological study and was capable of estimating whether or not participants had consumed wine in the previous 3 days (13).

Multi-biomarker panels can be applied to classify and monitor adherence to dietary patterns. This was recently illustrated in post-menopausal women where a biomarker panel was capable of discriminating between high and low quintiles of adherence to four diet scores [the alternate Mediterranean diet score (aMED), alternate Healthy Eating Index (AHEI)-2010, Dietary Approaches to Stop Hypertension (DASH) diet, and the Healthy Eating Index (HEI)-2015] (14). Another interesting application of multiple biomarkers is the interrogation of the relationships between food intake and diseases incidence. In this context previous work has employed a biomarker score, derived from multiple biomarkers of fruit and vegetable intake, as a proxy for intake to examine the relationship with diabetes incidence (15). This biomarker score demonstrated an inverse association with diabetes incidence, with odds ratio (OR) of incidence decreasing with increasing intake of fruit and vegetables (highest quartile of intake compared to lowest OR: 0.13; 95% CI: 0.08, 0.21) (15). Collectively, these studies highlight the potential for combinations of multiple biomarkers in determining intake of foods or dietary patterns and assessment of relationships with health outcomes.

Fruit is an important component of a healthy diet and previous work has investigated biomarkers of various fruit. A recent systematic review of the literature conclude that we have limited knowledge for biomarkers of pome and stone fruit (16) with many biomarkers requiring validation in terms of relating to fruit intake. With respect to apple intake the most promising biomarker identified was phloretin and phloretin glucuronide. The biomarker arbutin is promising for pear intake but again requires more validation. A review of biomarkers of tropical fruit intake also highlighted a dearth of information (17), concluding clearly that there is a need for further research in the area. Proline betaine is a well-established biomarker of citrus intake with previous work indicating that urinary proline betaine concentrations can give quantitative food intake information (18, 19). However, as most consumers eat more than one fruit, there is an interest to examine total fruit intake and thus combining multiple biomarkers could be a useful approach to estimate total fruit intake.

Although previous work has indicated the potential of multiple biomarkers in the form of multi biomarker panels, combining of biomarkers to give quantitative information on food intake is not trivial. Previous studies have highlighted the potential of multi biomarker panels in terms of classification into consumers and non-consumers. However, more work is needed in the field to examine the potential of such biomarker combinations for use in assessment of consumption of different quantities of food. Therefore, the objective of this work was to examine the ability of a multi-biomarker panel to classify individuals into categories of fruit intake.

## Materials and Methods

### National Adult Nutrition Survey (NANS) Study—Cross-Sectional Study

Details of the NANS study have been published elsewhere (https://www.iuna.net/) (20). Ethical approval for this study was granted by the University College Cork Clinical Research Ethics Committee of the Cork Teaching Hospitals [ECM 3 (p) 4 September 2008] and recruitment began in May 2008. Briefly, NANS collected data on habitual food and beverage consumption, lifestyle, health indicators, and attitudes to food and health in 1,500 adults, representative of the population during 2008–2010 in Republic of Ireland. A subset of this population (*N* = 565) was randomly selected, to ensure equal numbers of men and women across the age ranges (18–90 years) for metabolomics analysis as previously described (20). A 4 days semi weighed dietary record was used to collect dietary data over 4 consecutive days. Detailed information on the type and amount of all foods, drinks and nutritional supplements consumed over the 4 days was recorded by participants. Where possible, participants were asked to weigh foods and encouraged to retain food packaging for further information on foods consumed, but where weights were not recorded a photographic food atlas was used to estimate food weights (18). Dietary data was analyzed using WISP software (Tinuviel Software, Anglesey, UK), which uses data from McCance and Widdowson's The Composition of Foods, fifth and sixth editions and all supplementary editions to generate nutrient data (21–24). Dietary data was coded into 2552 individual food codes and grouped into one of 68 food groups. For the purpose of this analysis any fruit containing food groups were collapsed into a single “Total Fruit Intake” group and the mean daily fruit intake over the 4 days was calculated. Biological samples were collected at the end, or as close to as possible, of the dietary recording period, including a fasting first void urine sample in a sterile 50 mL tube which was chilled until processing. All urine samples were centrifuged at 1,800 × g for 10 min at 4°C. Aliquots of 1 mL were stored at −80°C for later analysis.

### A-Diet Validation Intervention Study

Ethical approval for this A-Diet study was granted by the UCD Sciences Human Research Ethics Committee (LS-17-16-Brennan). Recruitment commenced at the end of March 2017 via advertisement (posters, flyers, and emails) and finished in November 2017. The recruitment process is outlined in Supplementary Figure 1. Details of the study design are published elsewhere (25). Briefly, inclusion criteria included healthy, non-pregnant/lactating and non-smoking individuals, between 18 and 60 years old, and with a body mass index (BMI) between 18.5 and 30 kg/m^2^, without any diagnosed health condition (chronic or infectious diseases), no consumption of medications/nutritional supplements or any allergies/intolerances to the test foods. Once informed consent was acquired, participants were assigned to either a lunch (*N* = 27) or dinner (*N* = 34) test meal group and invited to partake in a 5-weeks study. Each test week participants were provided with four portions of a test meal and asked to consume this test meal for 4 consecutive days. During these 4 days, participants were also asked to avoid consuming any other foods related to the test meal ingredients. Participants were also asked to record a 4 days dietary record for each test week, to ensure compliance. The focus of this analysis is the amount of fruit consumed as part of these test meals. The low, medium, and high portions of fruit provided was 50, 100, 300 g and 80, 160, 320 g for apples and oranges, respectively.

Fasting first void urine was collected after an overnight 12 h fast at the end of each test week and chilled until processing. All urine samples were centrifuged at 1,800 × g for 10 min at 4°C. Aliquots of 1 mL were stored at −80°C for later analysis.

### Metabolomic Analysis of Urine Samples

Metabolomic analysis was performed using nuclear magnetic resonance (NMR) spectroscopy. Urine samples were first defrosted and then prepared by addition of 250 μL phosphate buffer (0.2 mol KH_2_PO_4_/L, 0.8 mol K_2_HPO_4_/L) to 500 μL urine. After centrifugation at 5,360 × g for 5 min at 4°C, 10 μL sodium trimethylsilyl [2,2,3,3-2H_4_] propionate (TSP) and 50 μL deuterium oxide (D_2_O) were added to 540 μL of the supernatant. Spectra were acquired on a 600 MHZ Varian Spectrometer (Varian Limited, Oxford, United Kingdom) by using the first increment of a nuclear Overhauser enhancement spectroscopy pulse sequence at 25°C. Spectra were acquired with 16,384 data points and 128 scans. Water suppression was achieved during the relaxation delay (2.5 s) and the mixing time (100 ms). All ^1^H NMR urine spectra were referenced to TSP at 0.0 parts per million (ppm) and processed manually with the Chenomx NMR Suite (version 7.7) by using a line broadening of 0.2 Hz, followed by phase and baseline correction. Three metabolites were chosen to demonstrate the proof-of-concept that combinations of biomarkers could be used to predict total fruit intake. The biomarkers chosen were xylose, proline betaine, and hippurate. Identification and quantification of metabolites was achieved using the Chenomx library. To confirm correct assignment, a urine sample was spiked with an analytical standard and a ^1^H NMR spectrum acquired.

Osmolality was measured by using an Advanced Micro Osmometer model 3300 (Advanced Instruments). Aliquots of urine were measured for osmolality with the use of micro-osmometry by freezing point depression, with values reported as the number of solute particles, in moles, dissolved in a kilogram of urine (mOSM). Metabolite concentrations were normalized to osmolality for quantifying urinary concentrations of xylose, proline betaine and hippurate.

### Statistical Analysis

Statistical analysis was performed using IBM SPSS software package version 24.0 for windows (SPSS Inc. Chicago, IL, USA) and SIMCA-P software (version13; Umetrics). One-way analysis of variance was performed to compare tertiles of self-reported intake and urinary concentrations of three biomarkers. Spearman's correlations were used to assess association between mean daily self-reported total fruit intake and biomarkers. The urinary concentrations of each biomarker were summed together to create a single combined biomarker value for each individual. Using the intervention data cut-offs were developed for certain fruit intake categories.

## Results

### Examining Relationship Between Biomarkers and Self-Reported Fruit Intake

Urinary biomarkers of interest were quantified by ^1^H NMR analysis in the NANS cross-sectional study. The following concentrations were obtained: xylose (range: 0.07–2.19 mM), proline betaine (range: 0.04–2.13 mM), and hippurate (range: 0.13–15.87 mM). Participants were grouped into tertiles based on self-reported mean daily intake of total fruit from semi-weighed food records (Table 1, Supplementary Table 1). All three urinary biomarker concentrations increased as the intake of fruit increased with significant increases observed for proline betaine and hippurate. The urinary concentrations of all three biomarkers were also significantly correlated with mean daily intake of fruit. The main contributors to total fruit intake were apples, bananas, oranges, pears, strawberries, and pineapple, all of which increased significantly across tertiles of intake except for pineapple.

**Table 1:** Urinary food intake biomarker concentrations across tertiles of fruit intake in cross-sectional study (NANS).

	**Tertile 1 (*****N*** **=** **180)**		**Tertile 2 (*****N*** **=** **181)**		**Tertile 3 (*****N*** **=** **185)**			**Spearman's correlation**^**+**^	
	**Mean**	***SD***	**Mean**	***SD***	**Mean**	***SD***	***p*-value**	**Rho**	***p*-value**
**Metabolite (μM/mOsm/kg)**									
Xylose	0.596	0.34	0.614	0.35	0.637	0.42	0.574	0.078	0.070
Proline betaine	0.285	0.22	0.415	0.34	0.569	0.44	<0.001	0.400	<0.001
Hippurate	3.600	2.78	3.991	2.87	4.727	3.20	0.001	0.181	<0.001
**Mean daily intakes (g/d)**									
Total fruit	28.42	24.61	127.80	32.61	315.19	115.44	<0.001		
Apples	4.94	13.50	23.93	30.76	58.15	65.78	<0.001		
Banana	7.17	15.28	26.86	31.77	43.07	42.70	<0.001		
Oranges	2.49	9.86	29.56	42.55	94.14	105.97	<0.001		
Pears	0.52	3.70	4.46	15.61	22.18	47.46	<0.001		
Strawberry	1.03	5.41	2.48	9.34	4.44	13.40	0.005		
Pineapple	0.47	3.73	1.20	7.47	2.75	16.91	0.129		

All values presented are mean and standard deviation (SD) unless stated otherwise. ANOVA, analysis of variance; MDI, Mean daily intake. N = 546.

+ *Spearman's correlation between each biomarker and total mean daily fruit intake*.

Further analysis was performed using total fruit consumers only (*N* = 509) (Table 2). Similar trends were observed for the urinary biomarker concentrations, increasing across of tertiles of intake with significant values for proline betaine and hippurate.

**Table 2:** Urinary food intake biomarker concentrations across tertiles of fruit intake in cross-sectional study (NANS), total fruit consumers only.

**Mean daily intakes (g/d)**	**Tertile 1 (*****N*** **=** **168)**		**Tertile 2 (*****N*** **=** **168)**		**Tertile 3 (*****N*** **=** **173)**		**One-way ANOVA**	**Spearman's correlation**^**+**^	
	**Mean**	***SD***	**Mean**	***SD***	**Mean**	***SD***	***p*-value**	**Rho**	***p*-value**
**Metabolite (μM/mOsm/kg)**									
Xylose	0.601	0.35	0.604	0.35	0.649	0.43	0.424	0.090	0.043
Proline betaine	0.298	0.24	0.425	0.33	0.581	0.45	<0.001	0.382	<0.001
Hippurate	3.622	2.63	3.957	2.82	4.771	3.24	0.001	0.187	<0.001
**Mean daily intakes (g/d)**									
Total fruit	42.67	26.44	139.58	32.28	323.36	114.98	<0.001		
Apples	8.66	18.54	25.60	33.86	59.09	66.35	<0.001		
Banana	10.05	17.90	29.61	32.63	43.12	43.46	<0.001		
Oranges	4.18	13.92	33.34	43.80	97.75	108.18	<0.001		
Pears	1.11	6.39	4.24	15.46	23.71	48.71	<0.001		
Strawberry	1.36	6.01	2.42	9.45	4.74	13.81	0.008		
Pineapple	1.07	7.08	1.14	7.21	2.55	16.75	0.399		

All values presented are mean and standard deviation (SD) unless stated otherwise. ANOVA, analysis of variance; MDI, Mean daily intake. N = 509.

+ *Spearman's correlation between each biomarker and total mean daily fruit intake*.

### Using Multiple Biomarkers to Classify Individuals Into Categories of Intake

The multi-biomarker panel was used to create a combined biomarker value by summing the individual biomarkers. In the A-Diet validation dataset (*N* = 160) the combined biomarker values were used to determine cut-offs for classification of individuals into categories of intake. Participants were categorized into one of three groups of intakes (0–100, 101–160, and >160 g/d) based on the fruit consumed as part of the intervention. The average sum of biomarkers for participants who consumed ≤ 100 g/day of fruit (4.766 μM/mOsm/kg) was set as cut-off point 1. Cut-off 3 was calculated as the average biomarker sum (5.976 μM/mOsm/kg) of participants who consumed >160 g of fruit in the A-diet study. The middle cut-off was set at any value in between cut-off 1 and 3 (Table 3).

**Table 3:** Cut-off points for each of the fruit intake categories derived from the Intervention Study.

**Fruit intake category (g/d)**	**Biomarker cut-offs (μM/mOsm/kg)**
0–100	<4.766
101–160	4.766–5.974
>160	≥5.975

*Cut-offs were calculated using sums of biomarker concentrations in each intake category from the A-Diet intervention study*.

To examine the ability of these biomarker sum values to categorize participants into categories of intake the method was applied to NANS study. Using the NANS (*N* = 546) self-reported dietary data participants were categorized into three groups of fruit intake (Table 4). Independently using the urinary biomarker concentrations, participants were assigned to a category of intake using the biomarker sum cut-offs. Excellent agreement between the two methods was observed with for example, 97 participants self-reporting intakes between 101 and 160 g/d, and 86 participants assigned into this category based on the biomarker panel data (Table 4 and Supplementary Table 2). Assessment of the data split by gender revealed similar agreement trends (Table 5).

**Table 4:** Classification into categories of fruit intake based on biomarker data or based on self-reported intake data in the cross-sectional study (NANS).

**Fruit intake category (g/d)**	**Self-reported data (N)**	**Biomarker data (N)**
0–100	227	306
101–160	97	86
>160	222	154

*Participants were classified into one of 3 categories based on either (1) Self-reported dietary data or (2) biomarker data. The number of participants into each category is reported, N = 546*.

**Table 5:** Distribution of participants classified into each category of intake based on sum of urinary biomarker concentrations compared to reported intake data in the cross-sectional study, split by gender.

	**NANS**			
	**Self-reported**		**Predicted**	
**Gender**	**M**	**F**	**M**	**F**
*N*	278	268	278	268
**Intake category**				
0–100 (g/d)	128	99	170	136
101–160 (g/d)	42	55	42	44
>160 (g/d)	108	114	66	88

*Participants (N = 546) categories based on cut-offs were calculated using sums of biomarker concentrations in each intake category from the A-Diet intervention study. ADV, A-Diet Validation Study; NANS, National Adult Nutrition Survey; M, Male; F, Female; N, Number of participants*.

In order to examine other biomarker combinations, the biomarker values that increased across categories of intake were selected (Supplementary Table 3). The ability to categorize participants into categories of intake was examined with all biomarkers and the sum of proline betaine and hippurate providing the best agreement (Table 6).

**Table 6:** Urinary biomarker classification of fruit intake compared to reported intake in the cross-sectional study (NANS).

**Classification method**	**Self-reported intake**	**Sum of proline betaine and hippurate**	**Sum of xylose and proline betaine**	**Sum of all biomarkers**
**Average fruit intake (g/d)**	***N***	***N***	***N***	***N***
0–100	227	304	306	306
101–160	97	70	194	86
>160	222	172	46	154

*Number of participants (N = 546) classified into each intake category was based self-reported intake and on combinations of urinary biomarker concentrations*.

## Discussion

This study developed a biomarker panel that was capable of classifying individuals into categories of fruit intake. Examination of the approach in a free-living cross-sectional study revealed excellent agreement with self-reported intake. Similar number of participants were ranked into each category of fruit intake. The work illustrates the potential of multi-biomarker panels and paves the way forward for further development in the field.

The three biomarkers chosen to build the multi-metabolite panel in this research have frequently been found in previous research to be associated with fruit intake. Xylose was previously identified as a food intake biomarker associated with apple intake and capable of ranking participants in an observational study by increasing intake (25). Proline betaine is a well-established, robust, and quantitative biomarker of citrus intake (18, 26). Proline betaine was previously included as part of a biomarker panel for the investigation of orange juice intake and juice quality (27). Hippurate, has often been associated with consumption of polyphenol rich plant foods, such as citrus fruit, bananas, and berries (10, 26, 28–30). This research combined these three food intake biomarkers into a biomarker score for the successful ranking of fruit intake. It is important to acknowledge that there are other potential biomarkers of fruit intake; however, examination of the full panel of potential biomarkers was beyond the scope of the current work where the emphasis was on the demonstration of the potential of combination of biomarkers.

Previous multi-metabolite panels have focused on associating the panels with intake of certain foods or achieving a dichotomous classification of consumers and non-consumers. An example of previous work demonstrated good associations between biomarkers of fruit and vegetables intake and self-reported dietary records (12). When 24 h urinary concentrations of isorhamnetin, hesperetin, naringenin, kaempferol, and phloretin were combined in a panel they were correlated with fruit intake (*r* = 0.27, *p* = 0.06), fruit juice intake (*r* = 0.28, *p* = 0.04), and intake of total fruits and fruit juices (*r* = 0.38, *p* = 0.006). The correlations for the fruit related panel improved when examined in spot urine samples: fruit intake (*r* = 0.34, *p* = 0.01), fruit juice intake (*r* = 0.44, *p* = 0.001), and total fruit and juice intake (*r* = 0.47, *p* = 0.0004). The authors concluded that this combination of flavonoids could be used as a reliable biomarker of total fruit and juice intake, however, to the best of our knowledge there is no demonstration that the biomarkers could predict intake. Our work is an important advancement as it clearly demonstrates that the multi-biomarker approach is capable of a classification of intake into a range of categories. However, it should be noted that the present work did not make the distinction between whole fruit and fruit juices and the biomarker panel classification was based on total fruit intake including juices.

Our previous work identified four food intake biomarkers of sugar sweetened beverages (formate, citrulline, taurine, and isocitrate) using heat-map analysis of metabolomic urinary profiles from the NANS study (31). These markers were confirmed by food analysis of the sugar-sweetened beverage and an acute intervention study. The markers were combined in a panel and ROC curves demonstrated that the panel could discriminate between consumers and non-consumers of sugar-sweetened beverages (AUC = 0.8) and was more predictive of intake than the individual biomarkers themselves (AUCs ranging from 0.5 to 0.7). A recently published study used data from the KarMeN study to identify five metabolites [methoxyeugenol glucuronide (MEUG-GLUC), dopamine sulfate (DOP-S), salsolinol sulfate, 6-hydroxy-1-methyl-1,2,3,4-tetrahydra-β-carboline sulfate, and xanthurenic acid] that were discriminative between high and non-consumers of banana (10). Individually, DOP-S had the best prediction ability [AUC = 0.84, error rate (ER) = 0.25] for classifying high consumers against non-consumers but was not as robust as a combination of all five metabolites (AUC = 0.90, ER = 0.13). However, the best predictive ability was a combined panel of MEUG-GLUC and DOP-S with the lowest error rate of misclassification. This research demonstrates how a panel of food intake biomarkers which individually were not robust enough, when combined can be used to classify recent banana intake. Our research takes the application of biomarker panels a step further by classifying participants into categories of fruit consumed and moves beyond the dichotomous classification of consumer/non-consumer.

While the above work demonstrates the potential for multi-biomarkers in terms of estimating food intake such panels can also be used to assess the relationship between food and diet-related diseases. A combined biomarker-score was developed using the standardized plasma values of vitamin C, β-carotene, and lutein, all of which were previously related to fruit and vegetable intake (15). This score was inversely associated with odds of incidence of type 2 diabetes (OR: 0.13; 95% CI: 0.08, 0.21) even after adjustment for lifestyle factors and demographics. An identified plasma biomarker panel representative of dietary habits consisted of β-alanine (beef intake), alkylresourcinols (wholegrain/rye), eicosapentaenoic acid and 3-carboxy-4-methyl-5-propyl-2-furanpropanoic acid (fish), lauric acid (saturated fats), linoleic acid (seeds, nuts, and vegetable oils), oleic acid (olive and rapeseed oil), and α and γ tocopherol (32). This combined panel was capable of predicting new cases of type 2 diabetes over a 5-years follow-up period with a specificity and sensitivity similar to classic diabetes predictors (serum adiponectin, insulin resistance, impaired glucose tolerance and impaired fasting glycaemia). Collectively, these and other studies highlight the potential of a multi-metabolite panel for the assessment of the relationship between diet and health/disease.

If future studies develop comprehensive and validated multi-biomarker panels, they could add value to the assessment of dietary intake by enabling the capture of a broad range of dietary exposures including bioactive compounds, foods, food groups, and complex dietary patterns. Panels could then be used in epidemiological research to elucidate the mechanisms and metabolic pathways of diet-related diseases and to validate self-reported dietary data. Further development of more comprehensive panels could enable measurement of adherence to specific dietary patterns, such as the Mediterranean diet. Future challenges for the field will be finding the simplest combination of metabolites to accurately determine exposures as well as validating these panels to a standard where they can be applied in nutritional research and public health surveys (9). As the field develops further, there will be a need to develop new statistical tools to integrate multiple biomarkers with self-reported data. Our work has recently developed calibration equations based on biomarker-predicted citrus intakes to gain a more accurate and objective measure of true intake (33). Using biomarker data to correct self-reported data for food intake is a promising option and could be adapted to include a biomarker panel. Further work is to develop the statistical tools to achieve this.

This study has strengths and limitations. A limitation worth noting is the fact that we examined the ability of the biomarker panel to categorize fruit intake in a cross-sectional study where intake was estimated with self-reported data. Future studies where comparison is performed in large intervention studies would be useful to examine relationships with true food intake. On the other hand, including the development of a food intake biomarker panel with testing in cross-sectional study is a strength as it demonstrates that the panel was capable of ranking fruit intake at a population level, against the background of exposure to various other foods. This research did not examine the potential impact of gut microbiome on the biomarker panel. Future work is warranted to address this.

To conclude, this study successfully demonstrated the utility of a panel of biomarkers for estimating fruit intake. The identification of comprehensive and validated multi-biomarker panels related to certain foods will be important as this field develops. The use of such panels may be key to distinguishing foods and adding specificity to the predictions of food intake. Combining such panels with self-reported measures will be important for increasing the accuracy of dietary assessment methods. Furthermore, there is potential for the use of such panels in large epidemiological studies to examine the relationships between diet and disease.

## Data Availability Statement

The original contributions presented in the study are included in the article/Supplementary Material, further inquiries can be directed to the corresponding author/s.

## Ethics Statement

The studies involving human participants were reviewed and approved by UCD HREC. The patients/participants provided their written informed consent to participate in this study.

## Author Contributions

AM conducted the A-Diet Validation study, acquired, analyzed the data, and prepared the manuscript. LB designed the research, analyzed the data, and prepared the manuscript. BM, AN, JW, and AF contributed the data from the NANS study. All authors read and accepted the final version of the manuscript.

## Conflict of Interest

The authors declare that the research was conducted in the absence of any commercial or financial relationships that could be construed as a potential conflict of interest.
