# Supplementary material for: The Potential of Multi-Biomarker Panels in Nutrition Research: Total Fruit Intake as an Example
Source: Front Nutr. 2021 Jan 14;7:577720. doi: 10.3389/fnut.2020.577720 (PMC7840580; doi:10.3389/fnut.2020.577720)
Supplement: Supplementary file 1 [file Data_Sheet_1.docx]

**
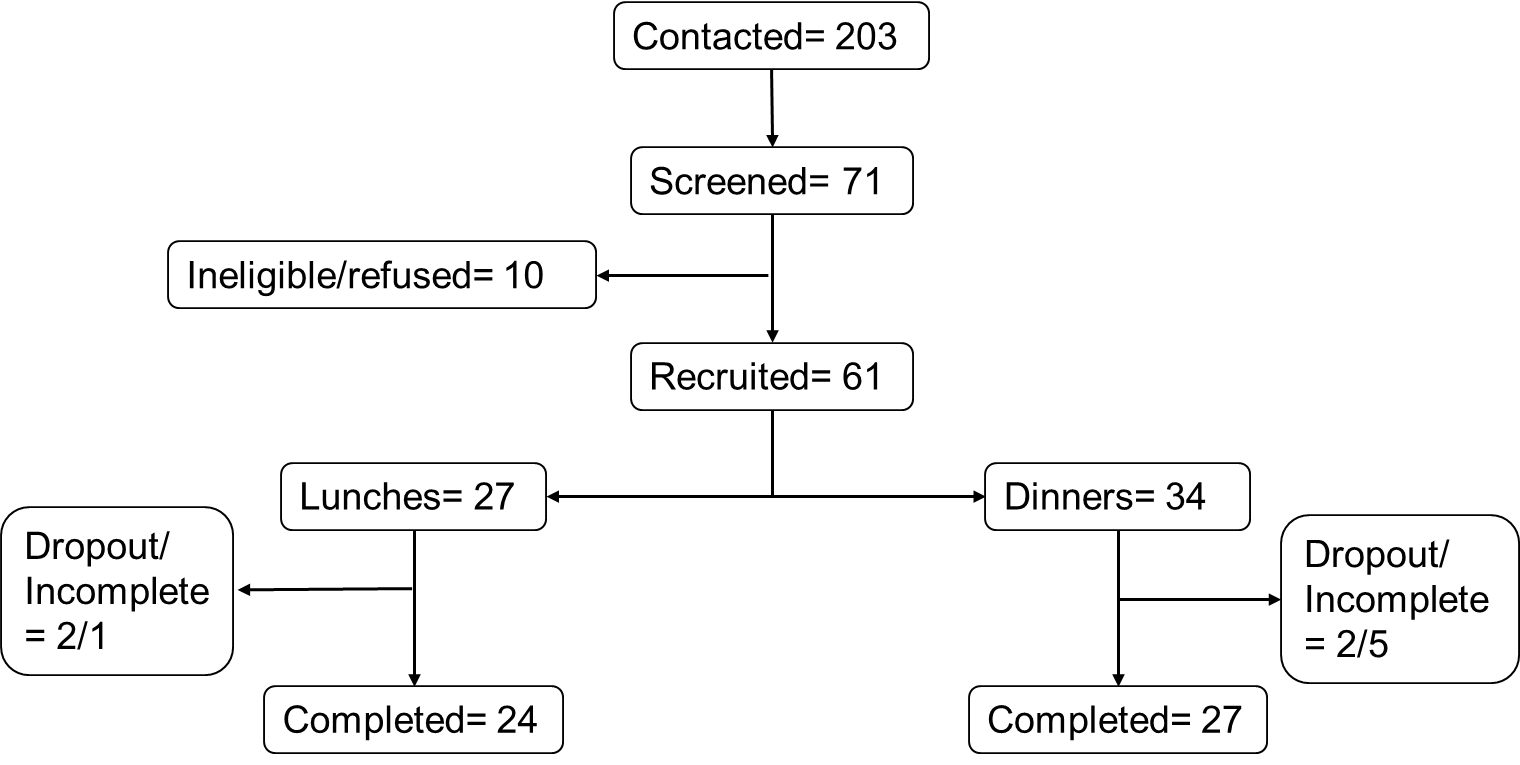
**

**Figure 1.** Outline of recruitment procedure for the A-Diet study.

**Supplementary Table 1.** Demographics of the intervention study population.

| **Intervention study** |  |
| --- | --- |
| **Characteristics** | (N=57) |
| **Gender** | 24M 33F |
| **Age (years)** | 27.89 ± 9.8 |
| **BMI (kg/m^2^)** | 23.34 ± 2.74 |
| **Cross-sectional study** | (N=546) |
| **Gender** | 278M 268F |
| **Age (years)** | 47.30 ± 16.5 |
| **BMI (kg/m^2^)** | 27.50 ± 5.15 |

**Supplementary Table 2.** Number of participants classified into each fruit intake category by both sum of urinary biomarkers cut-offs and self-reported fruit intake in the NANS cross-sectional study.

| **Amount of fruit consumed** |  |  | **Self-Reported** |  |
| --- | --- | --- | --- | --- |
| **Biomarker-Calculated** |  | **0-100 g/d** | **101-160** **g/d** | **>160 g/d** |
| **0-100 g/d** |  | 153 | 52 | 101 |
| **101-160 g/d** |  | 27 | 20 | 39 |
| **>160 g/d** |  | 47 | 25 | 82 |

Participants were classified into each intake category based on urinary biomarker concentration cut-offs (N=546). The cross-tabulation against self-reported fruit intake reveals how urinary biomarker cut-offs ranked individuals within each intake category.

**Supplementary Table 3**. Normalized urinary concentration of each biomarker and sum of urinary biomarkers across the three fruit intake categories in the intervention study.

| **Amount of Fruit** | **≤100g/d** | | **101-160g/d** | | **>160g/d** | |
| --- | --- | --- | --- | --- | --- | --- |
|  | **Mean** | **SD** | **Mean** | **SD** | **Mean** | **SD** |
| **Xylose** | 0.63 | 0.32 | 0.59 | 0.18 | 0.99 | 0.69 |
| **Proline Betaine** | 0.33 | 0.21 | 0.95 | 0.40 | 0.83 | 0.74 |
| **Hippurate** | 3.80 | 2.40 | 3.56 | 1.98 | 4.16 | 1.99 |
| **Sum of Proline Betaine and Hippurate** | 4.13 | 2.39 | 4.51 | 2.00 | 4.99 | 2.27 |
| **Sum of Xylose and Hippurate** | 4.44 | 2.52 | 4.15 | 1.98 | 5.15 | 2.21 |
| **Sum of Xylose and Proline Betaine** | 0.96 | 0.36 | 1.53 | 0.49 | 1.82 | 0.80 |
| **Sum of all biomarkers** | 4.77 | 2.50 | 5.10 | 2.01 | 5.98 | 2.38 |

Biomarker concentration values are presented as mean and standard deviation, (µM/ mOsm/kg).
